# A modified detachable snare closure for post-endoscopic submucosal dissection defects: the clip, loop, and clips technique

**DOI:** 10.1055/a-2566-9462

**Published:** 2025-04-09

**Authors:** Hiroki Hayashi, Hisashi Fukuda, Jun Owada, Haruo Takahashi, Yuji Ino, Ayman Qawasmi, Hironori Yamamoto

**Affiliations:** 1 12838 Department of Medicine, Division of Gastroenterology, Jichi Medical University, Shimotsuke, Japan; 2 Department of Medicine, Division of Gastroenterology, Samson Assuta Ashdod University Hospital, Ashdod, Israel; 3 Department of Medicine, Division of Gastroenterology, Jichi Medical University, Tochigi, Japan

Endoscopic closure of mucosal defects following gastric endoscopic submucosal dissection (ESD) using an endoloop and endoclips has been shown to reduce post-ESD bleeding, particularly in patients receiving antithrombotic therapy 1 . Various endoloop closure methods have been proposed, but they often require specialized equipment, such as a dual-channel endoscope, limiting their applicability. The bead-assisted endoloop closure method (bead, loop, and clips [BLC]) introduced in 2022 2 facilitated closure with a single-channel endoscope, but posed challenges relating to foreign body insertion. In 2024, Tamaru et al. described the “clip and pull” method, which employs endoclips for endoloop manipulation without additional devices 3 . Building on these approaches, we propose the clip, loop, and clips (CLC) closure method, combining simplicity and efficiency using standard endoscopic tools.

The technique was used in a 78-year-old man undergoing ESD for a gastric lesion while on aspirin therapy. As shown in Fig. 1 , an endoclip (HX-610-090L; Olympus, Tokyo, Japan) was placed at the base of a disposable endoloop device (HX-400U-30; Olympus) and transported to the defect site using a re-openable clip (ROCC-F-26-195-C; MicroTech, Nanjing, China). The reopenable clip was placed on the anal side of the mucosal defect, and additional clips were placed circumferentially, anchoring the endoloop to the mucosal defect. The endoloop was subsequently tightened using biopsy forceps (Radial Jaw 4P; Boston Scientific, Boston, Massachusetts, USA) passed through its sheath, with the clip acting as an anchor, achieving complete closure ( Fig. 2 ). The procedure is detailed in Video 1 . Successful defect closure was confirmed by follow-up endoscopy the following day ( Fig. 3 ). There were no adverse events, in particular no post-ESD bleeding.

**Fig. 1:**
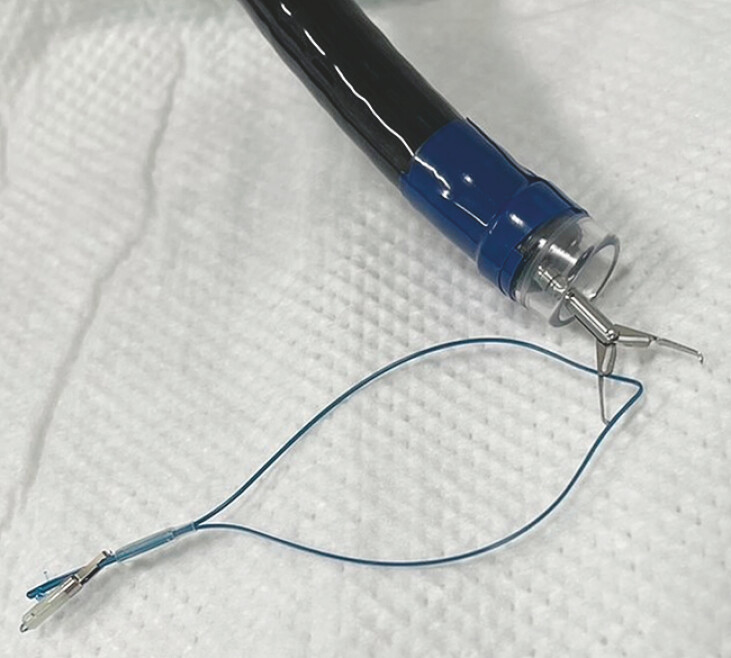
Photograph of the pre-prepared disposable endoloop, with an endoclip placed on the tail of the endoloop to act as an anchor and a 3–0 nylon thread tied to the tail of the endoloop to prevent migration of the clip.

**Fig. 2:**
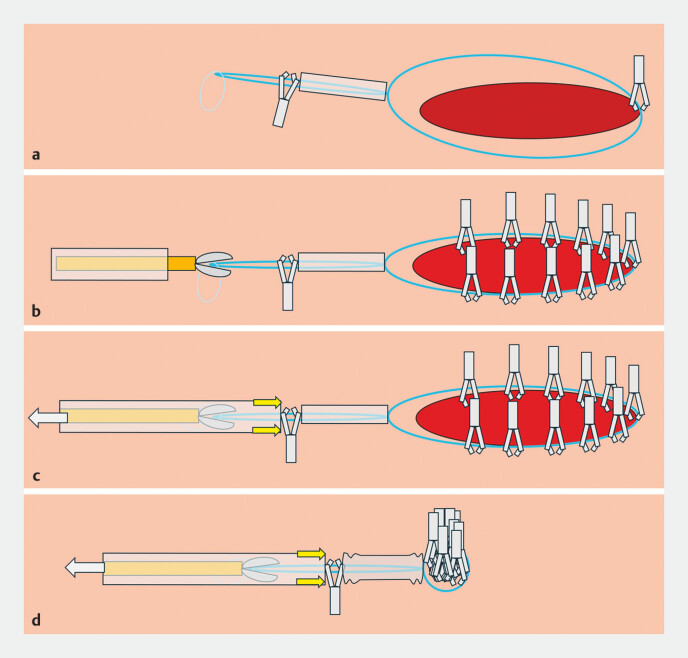
Schema of the clip, loop, and clips closure method showing: **a** the prepared endoloop transported to the defect site and fixed with a re-openable endoclip; **b** additional clips placed circumferentially to anchor the endoloop onto the mucosal defect, with the tail of the endoloop then grasped with a biopsy forceps passed through the outer sheath of the endoloop device; **c** the sheath being advanced by pulling the biopsy forceps; **d** the sheath and endoclip pushed against the loop stopper and the tightened endoloop.

Successful endoscopic closure using the clip, loop, and clips (CLC) closure method for a post-endoscopic submucosal dissection gastric mucosal defect.Video 1

**Fig. 3:**
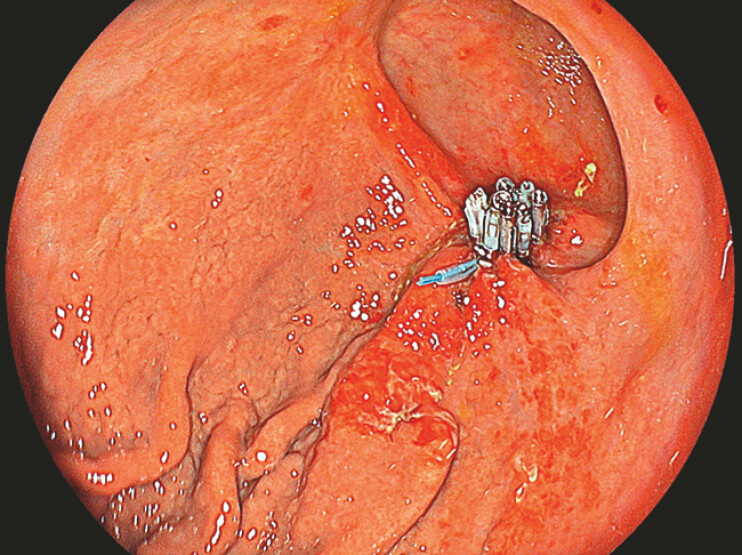
Follow-up endoscopy performed the following day showing successful defect closure, with no post-endoscopic submucosal dissection bleeding.

The CLC closure method represents a straightforward and effective approach, particularly for moderate-sized defects and high risk patients, that can be performed using standard endoscopic instruments.

Endoscopy_UCTN_Code_TTT_1AO_2AO

## Notes

Endoscopy E-Videos https://eref.thieme.de/e-videos
*E-Videos* is an open access online section of the journal *Endoscopy* , reporting on interesting cases and new techniques in gastroenterological endoscopy. All papers include a high-quality video and are published with a Creative Commons CC-BY license. Endoscopy E-Videos qualify for HINARI discounts and waivers and eligibility is automatically checked during the submission process. We grant 100% waivers to articles whose corresponding authors are based in Group A countries and 50% waivers to those who are based in Group B countries as classified by Research4Life (see: https://www.research4life.org/access/eligibility/ ). This section has its own submission website at https://mc.manuscriptcentral.com/e-videos .
